# Modulating mind-wandering in dysphoria

**DOI:** 10.3389/fpsyg.2013.00888

**Published:** 2013-11-27

**Authors:** Fionnuala Murphy, Kirsty Macpherson, Trisha Jeyabalasingham, Tom Manly, Barnaby Dunn

**Affiliations:** ^1^MRC Cognition and Brain Sciences UnitCambridge, UK; ^2^Department of Psychology, University of CambridgeCambridge, UK

**Keywords:** depression, cognition, sustained attention, rumination, mood, mind-wandering

## Abstract

Depression is associated with significant difficulty staying “in the moment” as the mind tends to wander away from current activity to focus instead on personal concerns. Mind-wandering (MW) may in some instances be a precursor for depressive rumination, a thinking style believed to confer vulnerability to the likelihood and extent of depression. Thus, MW may be not only a consequence but also a cause of low mood. Identifying a paradigm that could modulate MW, particularly in depressed individuals, would allow future studies to test whether elevated rates of MW causally drive cognitive-affective features of depression, such as rumination and anhedonia. This study therefore explored the feasibility of using an existing task manipulation to modulate behavioral and self-report indices of MW in participants with varying levels of self-reported dysphoria. Participants completed two go/no-go tasks—the SART and a high target probability task—and measures of state and trait MW. The two tasks were identical in all respects apart from the lower probability of no-go targets on the SART, a feature considered to encourage mindless, or inattentive, responding. Across participants, errors of commission (a behavioral indicator of MW) were elevated on the SART relative to the high probability task, a pattern that was particularly pronounced in dysphoric participants. Dysphoric individuals furthermore reported elevated levels of MW, though the modulation of these subjective reports by task was present to a similar rather than greater extent in the dysphoric individuals. These findings provide encouraging preliminary support for the use of this paradigm as one that modulates MW in depressed individuals. The implications of these results and directions for future research are discussed.

## Introduction

Much research into cognitive processing in depression has focused on depressive rumination, a thinking style believed to confer vulnerability to depressive episodes and to exacerbate the course of depression (Nolen-Hoeksema and Morrow, 1993; Nolen-Hoeksema et al., 1993; Nolen-Hoeksema, 2000). The term rumination refers to a response style that is characterized by a persistent and repetitive focus on thoughts about the causes, meanings, and consequences of one's low mood (Nolen-Hoeksema, 1991). While much research has been devoted to the consequences of depressive rumination, there has been little consideration of the factors that may make rumination more likely. Given that attention has a limited capacity, embarking upon ruminative trains of thought, for example, must often entail disengagement of attention from the here-and-now toward what is often personally-relevant and negative material.

This process of disengaging from the present has been explored in different contexts and described by a number of overlapping terms. These include mind-wandering (MW) (Smallwood and Schooler, 2006), stimulus-independent thought (Teasdale et al., 1995), task-unrelated thought (TUT) (Smallwood et al., 2009), absent-mindedness (Robertson et al., 1997; Cheyne et al., 2006), and daydreaming (Klinger, 1990). This work has tended to focus on MW's adverse effects upon performance and cognition, in both laboratory and real-life settings. For example, MW has been associated with impaired comprehension during reading (e.g., Smallwood et al., 2008). However, it has been suggested that MW has an additional important functional role. This has been borne out in experimental studies showing that MW facilitates specific cognitive functions such as future planning and problem-solving (see Mooneyham and Schooler, 2013, for a review).

There is accumulating evidence for an association between MW and depressed or dysphoric mood. In an early study (Giambra and Traynor, 1978), subjective reports of daydreaming, or MW, were found to correlate with three measures of depression in depressed individuals. In another study of participants with a diagnosis of depression, lapses of attention in everyday life and in a laboratory-based reading test were closely related and typically due to MW, rather than to “blanking” or external distraction (Watts and Sharrock, 1985). A series of recent correlational studies furthermore demonstrated that the frequency of self-reported MW in everyday life is associated with higher levels of self-reported depression and state negative affect (Stawarczyk et al., 2012). Other studies have also reported links between dysphoric mood and MW (e.g., Smallwood et al., 2005, 2007). However, few studies have addressed causality in this relationship. A notable exception is a study by Smallwood et al. (2009) in which negative and positive mood were induced in the laboratory. Their findings demonstrated that relative to positive mood, negative mood resulted in an increase in the frequency of self-reported task-irrelevant thoughts and also more errors on what is often considered to be a behavioral index of MW derived from a laboratory test of sustained attention, the Sustained Attention to Response Task (SART).

In line with the role ascribed to depressive rumination above, there is emerging interest in the idea that MW and related processes are not only a consequence of, but also causal in the development and maintenance of low mood. An experience sampling study conducted in ~5000 unselected individuals across 83 countries linked MW to reduced levels of happiness during everyday life (Killingsworth and Gilbert, 2010). Whilst this association may have reflected an influence of momentary low mood on MW (e.g., via ruminative content) or alternatively, an influence of some other common factor (e.g., poor cognitive control) on both mood and MW, time-lag analyses suggested that, MW was the cause, and not merely the consequence, of low mood. It is worth noting that the Killingsworth and Gilbert study focused on the association between MW and state, rather than longer term trait, moods. Stawarczyk et al. (2013) have provided further evidence for the involvement of MW in maintaining state negative mood over time. In their study, induced anticipation of a negative event, relative to a neutral one, predicted MW episodes specifically related to induced concern, and this in turn predicted subsequent levels of momentary negative affect.

In conceptual terms, MW can be contrasted with “mindfulness.” Mindfulness is a form of cognitive stance characterized by “paying attention in a particular way: on purpose, in the present moment, and nonjudgmentally” (Kabat-Zinn, 1994). An example includes being able to maintain focus on the coming or going of one's breath, noting but having the ability to move on dispassionately from the occurrence of distressing thoughts. Another example includes being able to engage in a current task such that the task, rather than self-conscious reflection, dominates the mental landscape. The supposed benefits of practice in mindfulness have led to its adoption in some forms of cognitive therapy for depression, such as mindfulness-based cognitive therapy, or MBCT (Segal et al., 2002; Piet and Hougaard, 2011). Though the precise mechanisms of action of mindfulness remain poorly understood, MW and particular elements of mindfulness—such as paying attention in the present moment—can be considered opposing constructs. Mrazek et al. (2012) demonstrated negative correlations between a measure of dispositional mindfulness and four measures of MW. They also showed a reduction in MW on a test of sustained attention immediately following 8 min of mindful breathing, relative to either reading or passive relaxation.

Thus, there is increasing clinical interest in the idea that altered mood in depression may be maintained, at least in part, by difficulties keeping “in the moment” and that interventions that reduce, or enhance control over, MW could have a beneficial effect upon mood in depression. Finding ways to reduce MW, however, requires a better understanding of the factors that influence the likelihood that the mind will wander.

Basic research in healthy populations has identified dispositional and situational factors that influence the prevalence and extent of MW. For instance, levels of MW are higher in younger relative to older individuals (Jackson and Balota, 2012) and in those with a lower working memory capacity (McVay and Kane, 2009, 2011). Individuals also demonstrate more MW at slower stimulus presentation rates (Smallwood et al., 2004) and as a task becomes more familiar over longer task durations (Manly et al., 1999; Smallwood et al., 2003, 2004; McVay and Kane, 2009). These experimental manipulations have been only minimally applied in individuals experiencing depressed or low mood, and thus, whether these manipulations have similar effects in these individuals remains unknown.

Identifying a method that reliably influences rates of MW in depressed individuals could be of significant value in the mood and MW literature, particularly as existing evidence for a link between mood and MW is largely associative and correlational. A paradigm that modulates MW, particularly in depressed individuals, would allow experimental control over a process considered important in the development and maintenance of depressed mood. Such an approach would make it possible to test whether elevated rates of MW causally drive cognitive-affective features that are central to depression, such as rumination or anhedonia.

The current study thus aimed to determine whether a laboratory manipulation considered to encourage an automatic and “mindless” mode of responding to differing extents influences behavioral and self-report measures of MW, particularly in individuals experiencing naturally-occurring dysphoric mood. Individuals that varied in the degree to which they reported experiencing symptoms of depression, indexed via the Beck Depression Inventory (BDI-II) (Beck et al., 1996), completed two different versions of a go/no-go task that differed in the probability of no-go targets, a design feature that in addition to the continuous performance element has been argued to determine the duration over which participants must maintain active attention (Manly et al., 1999). Immediately following performance of each task, retrospective self-report measures of MW were administered so that the correspondence between behavioral and self-report measures of MW could be examined. These included a thought content scale that has been used previously to index the subjective experience of TUT and task-related interference (TRI) (e.g., Smallwood et al., 2004), along with scales intended to index the experience of MW to positive or negative themes.

One of the tasks was the SART, performance on which has been shown to correlate significantly with reports of everyday cognitive failures, or absendmindedness, in individuals who have sustained a traumatic brain injury (Robertson et al., 1997). In the SART, participants are presented with a regular, rhythmic sequence of single digits and are asked to make the same, single-button response to each with the exception of a nominated, infrequent (11%) no-go target (e.g., “3”). To avoid errors on the no-go trials, participants must maintain active attentive control over responding in order to resist the tendency to press to every digit (Manly, 1999; Smallwood et al., 2004; Smallwood and Schooler, 2006). It has been argued that the repetitive, relatively unselective, and frequent response can become rapidly automated, allowing participants to devote attentional resources to activities other than those exclusively focused on task completion, such as TUT or MW. The other task was a modified response withholding task in which participants are required to withhold a response to targets on a much higher proportion (50%) of no-go trials. Previous work has demonstrated that the SART discriminates between individuals that differ in the extent of everyday self-reported absentmindedness indexed via the Cognitive Failures Questionnaire (CFQ; Broadbent et al., 1982) whereas the high probability task does not (Manly et al., 1999). Inclusion of the modified response withholding task allows determination of whether any observed impairments are due to the requirement to maintain attentive control over responses or to more general features of the task, such as the requirement for inhibitory control.

Responses on no-go trials on the SART, that is, errors of commission, reflect, at least in part, the efficiency of this strategy, relating to questionnaire measures of absentmindedness and MW (Robertson et al., 1997; Manly et al., 1999; Smallwood et al., 2004; Smallwood and Schooler, 2006). A second key performance index is variability in response times (RTs) during the task. At either extreme of a theoretical performance distribution it might be expected that variation in RTs would be relatively small; a participant who responds to all trials in an inattentive manner may well do so entrained to the rhythmic presentation of the digits and show little variability. A participant who carefully scrutinizes each digit to establish whether a response is required may also show remarkably consistent reaction times. In contrast, a participant who lapses between periods of careful attention and of “absentminded” response production in which their mind is elsewhere is likely to show much greater variability overall. Consistent with this analysis, it has been suggested that the RT coefficient of variability (CV) may reflect subtle differences in RT that are due to lapses of attention, thus providing a good index of MW (e.g., McVay and Kane, 2009; Seli et al., 2012).

The SART has been employed as a behavioral proxy of MW as performance on this task has been shown to correlate with online and retrospective self-report measures of MW (Smallwood et al., 2004; Marchetti et al., 2012). Consistent with the elevated levels of subjectively-experienced MW associated with depression, there have been reports of performance difficulties on the SART in clinically depressed or dysphoric individuals. For instance, a study of UK military personnel found increased numbers of commission errors in depressed individuals (Farrin et al., 2003). However, the increase in commission errors associated with depression has rarely been linked to subjective indices of MW. Smallwood et al. (2004) collected performance data and measures of subjective MW on the SART, along with depression symptomatology, but the relationships between these indices and depression severity were not discussed. Stawarczyk et al. (2012) reported an association between increasing depression severity and reports of everyday MW (i.e., daydreaming) and also between everyday MW and MW during the SART but did not report SART errors or RTCV. The relationship between SART commission errors and subjective MW was recently indexed using intermittent thought probes in 23 students that varied on the BDI (Deng et al., 2012). Depression severity correlated negatively with subjective reports of being “on-task” and positively with reports of both MW without awareness and dispositional mindfulness, but not with SART commission errors. Considering these studies together, in individuals experiencing low mood, the relationship between behavioral and self-report measures of MW on the SART remains unclear.

Understanding the relationship between behavioral and self-report measures of MW and the conditions that modulate the extent to which the mind wanders in dysphoric individuals is important from both theoretical and clinical perspectives. With this in mind, the current study was implemented primarily to explore the feasibility of modulating MW, particularly in dysphoric individuals, using the laboratory manipulation described above. The specific aims of this study were nonetheless threefold. First, it aimed to investigate the extent of MW, measured both behaviorally and via self-report, in individuals with varying levels of dysphoria using the SART. Second, it aimed to examine whether a task manipulation can be used to modulate not only performance, but also the subjective experience of MW, such that “mindlessness” was more evident on the SART than on a task with a higher probability of targets, particularly in dysphoric individuals. Third, this study aimed to explore the extent to which behavioral performance on these tasks related to self reports of MW, dispositional mindfulness or “acting with awareness,” and to individual differences in rumination and everyday attention failures.

We based our hypotheses on predictions arising from neuropsychological investigations of cognitive function and sustained attention in depressed and brain-injured populations and on those arising from the burgeoning literature on MW and mood. First, we hypothesized that there would be more commission errors on the SART than on the high probability task across participants. Our second hypothesis was that our task manipulation would also influence retrospective reports of subjective MW during task completion such that these would be higher following the SART than following the high probability task. Third, we predicted that the differential effects of our task modulation on commission errors and possibly RTCV, as well as subjective indicators of MW, would be particularly pronounced in dysphoric, relative to nondysphoric, participants. Our fourth hypothesis was that errors on the low probability SART would correlate *positively* with subjective retrospective reports of MW, self-report measures of everyday attentional failures (ARCES), and trait rumination, and *negatively* with subjective reports of everyday mindfulness (AAS of the FFMQ).

## Materials and methods

### Participants

A community sample of 44 participants with varying levels of depression severity, indexed using the BDI-II, was recruited from the Medical Research Council Cognition and Brain Sciences Unit (MRC CBU) participant panel. One participant was set aside due to extremely high error *and* omission rates, reflecting a failure to understand the task instructions, leaving 43 (31 female) participants between 18 and 64 years of age. The mean BDI of this sample was 9.84 (*SD* = 9.36), with BDI scores ranging between 0 and 29. According to the recommended BDI-II cutoffs (Beck et al., 1996), two participants had mild (BDI between 14 and 19), seven participants had moderate (between 20 and 28), and 1 had severe depression (29 or greater). Premorbid verbal IQ was assessed using the National Adult Reading Test (Nelson and Willison, 1991). The study was approved by the local research ethics committee, and all participants provided written informed consent prior to taking part in the study. Participants received an honorarium of £6 per hour for their participation in the project. Demographic and mood characteristics are given in Table 1.

**Table 1:** **Demographic characteristics and mood measure data for study participants**.

	**All participants (*n* = 43)**	**Low BDI (*n* = 10)**	**High BDI (*n* = 10)**	**Low vs. High BDI comparison**
Age	38.44 (15.47)	35.60 (12.12)	42.80 (16.10)	*t*_(18)_ = −1.13, *p* = 0.27
FSIQ	113.10 (10.38)	111.88 (8.99)	112.74 (11.14)	*t*_(18)_ = −0.19, *p* = 0.85
BDI-II	9.84 (9.36)	0.20 (0.42)	24.75 (4.30)	*t*_(18)_ = −17.95, *p* < 0.001
RS	21.02 (5.54)	16.10 (5.00)	27.30 (2.58)	*t*_(18)_ = −6.29, *p* < 0.001
ARCES	33.44 (7.13)	30.80 (5.22)	39.90 (7.89)	*t*_(18)_ = −3.04, *p* < 0.01
AAS	18.16 (5.20)	21.90 (4.12)	12.10 (4.07)	*t*_(18)_ = 5.35, *p* < 0.001

Data are mean (SD).

SD, standard deviation; FSIQ, Full Scale IQ estimated using the National Adult Reading Test (NART); BDI, Beck Depression Inventory; RS, Short Response Styles Questionnaire; ARCES, Attention-Related Cognitive Errors Scale; AAS, Acting with Awareness Scale of the Five-Facet Mindfulness Questionnaire (FFMQ).

### Procedure

Participants attended a 90 min session during which they were seated in a comfortable chair in a quiet room. Participants completed the cognitive tasks and measures described below. Both the SART and high target probability tasks were completed by all participants, interleaved with a neutral filler task. The order in which the SART and high probability tasks were completed was delivered in a counterbalanced order. Several additional measures were also administered but are not reported here.

### Sustained attention to response task (SART; Robertson et al., 1997; Manly et al., 1999)

The SART is a computerized go/no-go task that was presented using ePrime software and requires participants to make a single button press to all stimuli except the designated no-go “target.” The digits 1–9 were the stimuli; the digit “3” was the target. These were presented in a quasi-random order for 250 ms, followed by a 900 ms mask comprised of a 29 mm diameter white ring with a white diagonal cross through it. The digits were presented centrally on a computer screen, in a white font against a black background. Five different font sizes (48, 72, 94, 100, and 120 point), corresponding to stimulus heights of 12–29 mm, were presented at random to reduce the likelihood of participants making responses based on perceptual templates of the stimuli and to encourage instead processing of the numerical values. Participants sat ~60 cm away from the computer monitor and were instructed to respond via button press to every digit (go trial) except “3” (no-go trial), giving equal weight to speed and accuracy. There were 270 trials presented in a continuous block lasting 5.2 min, with each of the digits from 1 to 9 (including the target “3”) presented 30 times, such that the probability of no-go targets was 0.11. The 270 test trials were preceded by 18 practice trials.

The critical SART measure is the number of no-to trials on which a participant fails to withhold a response (i.e., errors of commission). However, other performance measures include: (1) RT for correct responses on go trials (RT); (2) a measure of within-participant variability of RTs, such as the standard deviation (SD), or when mean RT and RT variance is positively correlated (Seli et al., 2012), the RT coefficient of variation (RTCV), which is computed by dividing the SD by the mean RT across all trials; and (3) failures to respond to go trials (i.e., omission errors).

### High target probability task (Manly et al., 1999)

The present study also included a modified response withholding task. This go/no-go task, also presented using ePrime software, was identical to the SART in all respects except that the frequency of the no-go target was very much increased. In this high probability variant, the target (“3”) was presented on half of the trials such that the target probability was 0.5. For the purpose of data analysis, it was considered necessary to equalize the number of targets in the two versions of the task. Thus, in the high probability task, 30 of the 135 target trials were designated for analysis in advance and at random, such that performance on these could be compared with the 30 target trials from the SART task.

### Filler task

To reduce the impact of carryover effects from performance and ratings on one variant of the SART to the other, participants viewed a brief 5-min neutral video in between of an individual talking about a neutral household task, e.g., hanging a picture.

### Thinking content scale of the dundee stress state questionnaire (DSSQ; Matthews et al., 1999, 2002)

This scale was used to index self-reported MW, retrospectively, immediately subsequent to completion of both the SART and the high probability tasks. The 16 items, which were embedded within the ePrime stimulus presentation software, required participants to indicate roughly how often they had had thoughts relating to each of a number of themes while performing the tasks, by choosing the appropriate number on a scale with endpoints 1 “never” to 5 “very often.” Participants reported the frequency of two types of subjective experience according to each of 2 eight-item factors (1) the experience of thoughts that are unrelated to what one is doing, that is, TUT (e.g., “I thought about personal worries” or “I thought about something that happened earlier today”), and (2) the experience of interfering thoughts regarding one's performance of the task, that is, TRI (e.g., “I thought about my level of ability” or “I thought about how much time I had left”). Of these two types of subjective experience, TUT is the index that is generally considered to correspond most closely to conceptualizations of MW (Smallwood et al., 2009; Stawarczyk et al., 2011). However, reports of TRI are also considered to be associated with reduced performance on the SART (Stawarczyk et al., 2011).

### Positive and negative mind-wandering rating scales

The specific nature of the TUT items of the DSSQ meant that this scale was unlikely to capture all incidences of MW experienced by our participants. Thus, immediately prior to completing the DSSQ items upon completion of the SART and high probability tasks, two prompts indexed the valence of participants' off-task thoughts: “I thought about something positive” and “I thought about something negative.” These prompts were presented following the general instruction (identical to that described above for the DSSQ) to indicate roughly how often participants had had each of the following thoughts while performing the task, by choosing the appropriate number on a scale from 1 “never” to 5 “very often.” Thus, these items indexed the extent to which participants' minds wandered to positive or negative themes while completing the tasks.

### National adult reading test—revised (NART; Nelson and Willison, 1991)

The NART is frequently used to estimate premorbid mental ability in patient and vulnerable groups as it contains 50 phonetically irregular words that require prior knowledge for their correct pronunciation. It is therefore considered to be resistant to the cognitive effects of psychopathology such as depression, providing a valid estimate of premorbid IQ (Crawford et al., 1987).

### Beck depression inventory—second edition (BDI-II; Beck et al., 1996)

The BDI-II is one of the most widely used instruments for measuring the severity of self-reported depression, or dysphoria. It contains 21 items that relate to mood (e.g., sadness and irritability), cognition (e.g., indecisiveness and guilt), and physical symptoms (e.g., changes in appetite and fatigue). Each item is rated on a 4-point scale with endpoints 0 and 3. The points for each item are summed to give a total BDI score, and it has been recommended that the severity of depression is indexed as follows: 0–13 = minimal depression; 14–19 = mild depression; 20–28 = moderate depression; and 29–63 = severe depression (Beck et al., 1996). The BDI-II is known to have strong psychometric properties, including high internal consistency and concurrent validity (Storch et al., 2004).

### Short response styles questionnaire (RS) (Treynor et al., 2003)

Depressive rumination was assessed using the 10 item RS. Items such as “I think, Why do I always react this way?” were rated on a 4-point scale with endpoints 1 “never” to 4 “always.” The short RS—which includes items that tap the key features of a ruminative response, such as focusing on one's negative emotional state, on self-evaluation, and on the causes and consequences of one's depressed mood—resulted from the removal from the 22 item Ruminative Responses Scale (RRS) of 12 items that were deemed similar to BDI items.

### Acting with awareness scale (AAS) of the five-facet mindfulness questionnaire (FFMQ; Baer et al., 2006)

This scale was employed to provide a measure of self-reported trait MW. Only the 8 items from the “acting with awareness” subscale were administered. Participants rated whether each item is generally true of them, on a scale ranging from 1 “never or very rarely true” to 5 “very often or always true.” The items assess the tendency to attend to one's activities in the moment as opposed to behaving on “automatic pilot” (e.g., “When I do things, my mind wanders off and I'm easily distracted”). In conceptual terms, the items measure MW but they are reverse scored so that each gives a measure of acting with awareness.

### Attention-related cognitive errors scale (ARCES) (Cheyne et al., 2006)

The ARCES is a brief self-report scale that measures the frequency of everyday performance failures for which an attention lapse or failure of sustained attention is the most likely cause. Cheyne et al. (2006) found that scores on the ARCES correlate significantly with commission errors on the SART.

## Results

For all analyses, alpha was set at *p* = 0.05, and the results of two-tailed tests are reported. Pearson correlations were initially computed to determine the relationships between various trait measures of mood and attention. To assess the influence of the probability of no-go target manipulation on behavioral and MW performance, data were assessed using paired *t*-tests or repeated-measures ANOVAs as appropriate. To examine whether these effects were further modulated by depressive symptomatology, the data were analyzed using two methods. First, data were analyzed continuously with mean-centered BDI scores entered into analyses as a covariate. This was considered appropriate as participants were recruited to provide a range of depressive symptoms. Second, data were analyzed on the basis of whether participants fell into a low or high BDI group. Previous work investigating groups of individuals that differed with respect to self-reported everyday cognitive failures and their performance on the SART selected individuals falling in the lower and upper quartile of the range for this characteristic across the entire sample (Manly et al., 1999). Adopting the same approach here, participants falling within the upper and lower quartile of BDI-II scores were selected such that 10 high and 10 low BDI participants were placed in the high and low BDI groups, respectively.

Whereas a median split on our sample would have placed a significant number of participants with minimal levels of depression in our “high BDI” group, the present approach ensured that all participants in this group minimally had mild self-report depression. While small, these sample sizes are in line with those employed in group analyses that have been described previously (Manly et al., 1999; O'Connell et al., 2008) and furthermore circumvent the difficulties inherent at moderate levels of depressive symptomatology where some participants with lower levels of BDI reported a history of depression whereas those with higher levels did not. Characteristics for the low and high BDI groups are given in Table 1. Importantly, these groups did not differ significantly with respect to age or NART FSIQ (both *p*s > 0.25). They did, however, differ markedly with respect to their scores on all of the questionnaires that indexed mood, everyday attention-related cognitive errors, and state MW: BDI-II [*t*_(18)_ = 17.95, *p* < 0.001], RS [*t*_(18)_ = 6.29, *p* < 0.001], ARCES [*t*_(18)_ = 3.04, *p* < 0.01], AAS [*t*_(18)_ = −5.35, *p* < 0.001].

### Relationship between different symptom measures

The relationships between the various mood and attention measures are presented in Table 2. In line with previous investigations, a strong correlation was observed between symptoms of depression indexed using the BDI-II and depressive rumination measured using the RS (*p* < 0.001). Furthermore, BDI-II scores correlated positively with the ARCES, a measure of self-reported attention-related cognitive errors (*r* = 0.57, *p* < 0.001) and negatively with scores on the acting with awareness scale of the AAS (*r* = −0.68, *p* < 0.001). Thus, as would be expected, individuals with greater levels of depression symptomatology reported making attention-related errors to a greater extent and reported acting with awareness to a lesser extent than those with reduced levels of depression symptoms.

**Table 2:** **Correlations between demographic and mood variables across the entire sample (*N* = 43)**.

	**RS**	**ARCES**	**AAS**
BDI-II	0.77^*^	0.57^*^	−0.68^*^
RS		0.50^*^	−0.63^*^
ARCES			−0.79^*^

BDI, Beck Depression Inventory; RS, Short Response Styles Questionnaire; ARCES, Attention-Related Cognitive Errors Scale; AAS, Acting with Awareness Scale of the Five-Facet Mindfulness Questionnaire (FFMQ);

* *p* < 0.001.

### Effects of target probability on SART performance

Behavioral data for the SART and high probability task are presented in Table 3. Our manipulation of the probability of no-go targets on the SART had the intended effect on task performance. Overall, participants made significantly more errors of commission (incorrect responses to no-go targets) on the SART relative to the high probability target task [*t*_(42)_ = 10.86, *p* < 0.001]. RTs were also affected by task, with significantly lower RTs on the SART relative to high target probability task [*t*_(42)_ = −9.35, *p* < 0.001]. Given the high correlation between mean RT and the variability of RTs (SD) [*r*_(43)_ = 0.62, *p* < 0.001], we analyzed the RTCV instead as it has been recommended as a more suitable measure under these circumstances (Seli et al., 2012). This was higher in the SART than in the high probability task [*t*_(42)_ = 2.29, *p* < 0.05]. Errors of omission (failing to respond on “go” trials) were very low and equated to approximately 0.8% of trials overall. As omission data violated the assumptions of parametric analysis, a Wilcoxon sign ranked test was conducted, but no effect of task was found (*p* = 0.33). Thus, consistent with previous reports (Manly et al., 1999), reducing the frequency with which participants were required to withhold responses to no-go targets—on tasks that were otherwise identical in terms of stimulus type and duration, inter-stimulus interval, number of trials, and required behavioral response—resulted in a significantly greater number of commission errors, quicker responses, and significantly more variable responding as indexed by the RTCV.

**Table 3:** **Behavioral performance and subjective experience data for all participants in the SART and high probability task**.

**College**	**SART**	**High probability task**
**BEHAVIORAL PERFORMANCE**		
Mean errors of commission	9.95 (4.89)	2.47 (2.27)
Mean errors of omission	1.98 (4.44)	2.07 (6.43)
RT	358.61 (73.18)	421.72 (56.77)
RTCV	0.26 (0.09)	0.23 (0.09)
**SUBJECTIVE EXPERIENCE**		
TRI	2.43 (0.77)	2.23 (0.71)
TUT	1.52 (0.47)	1.57 (0.59)
Positive MW	1.93 (0.94)	2.00 (1.00)
Negative MW	2.35 (1.15)	1.98 (0.94)

Data are mean (SD) values.

SART, Sustained Attention to Response Task; BDI, Beck Depression Inventory; RT, Response times; RTCV, RT coefficient of variation; TRI, task-related interference; TUT, task-unrelated thought; MW, mind-wandering.

### Effects of target probability on self-reported mind wandering

As described above, subjective experience during the SART and high probability task was indexed using the Thinking Content scale of the DSSQ. This was administered immediately following performance of both the SART and high probability task, providing retrospective and subjective measures of TRI and TUT while performing the task. As the specific items on the DSSQ may not have captured the full extent of experienced MW, and because the DSSQ does not specify the valence of these thoughts, additional scales were administered to index the extent to which participants' minds wandered to positive and negative themes.

A Two-Way repeated measures ANOVA was conducted on self-report data, with task (SART, high probability task) and thought content (TRI/TUT) as repeated measures. Though participants reported more TRI/TUT overall in the SART than in the high probability task (see Table 3), this main effect did not achieve significance [*F*_(1, 42)_ = 3.00, *p* = 0.09]. There was a significant effect of thought content [*F*_(1, 42)_ = 73.84, *p* < 0.001] which was due to participants reporting significantly more TRI than TUT overall (TRI: 2.33 ± 0.75; TUT: 1.54 ± 0.53). This effect was qualified by a significant interaction between task and thought content [*F*_(1, 42)_ = 7.59, *p* < 0.01]. TRI was more prevalent on the SART than on the high probability task [*t*_(42)_ = 10.34, *p* < 0.001] whereas TUT did not differ significantly across the two tasks (*p* = 0.34).

A Two-Way ANOVA with task (SART, high probability task) and MW valence (positive, negative) as repeated measures demonstrated a main effect of task [*F*_(1, 42)_ = 4.23, *p* < 0.05] and a significant task by valence interaction [*F*_(1, 42)_ = 4.99, *p* < 0.05]. A significant main effect of valence did not emerge [*F*_(1, 42)_ = 1.47, *p* = 0.23]. Thus, reports of positive/negative MW were higher following completion of the SART than that of the high probability task, an effect that appeared to be due to increased MW on negative themes. This finding will be discussed further, in relation to dysphoric symptoms, below.

### Influence of depressive symptomatology on task performance

With respect to behavioral performance on the two versions of the SART, there were two predictions. First, dysphoric mood was expected to have a negative impact upon SART performance, particularly in terms of the number of commission errors, but possibly also in terms of RT and RT variability. Second, any influence of depression on performance was expected to be more evident on the SART than on the high probability task (cf. Manly et al., 2004).

These predictions received partial support when commission error data were analyzed as a continuous design with task as a repeated measure (SART, high probability task) and mean-centered BDI as a covariate. The main effect of mean-centered BDI-II approached significance [*F*_(1, 41)_ = 3.73, *p* = 0.06], with a higher number of commission errors produced by high relative to low BDI participants. The interaction between BDI-II scores and task, however, did not [*F*_(1, 41)_ = 1.97, *p* = 0.17]. Thus, although participants made more SART errors on the SART relative to the high probability task (see above), and increasing self-reported depression symptomatology was associated with increasing numbers of commission errors overall, the difference in performance was not significantly modulated by symptom severity indexed continuously by the BDI-II.

**Figure 1:**
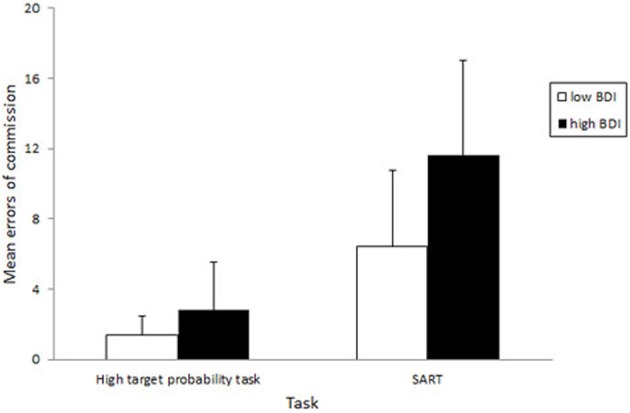
**Mean errors of commission on the SART and on a modified version of the task in low and high BDI participant groups**. Error bars are 1 SD.

In the group analyses, repeated-measures ANOVAs were employed with task (SART, high probability task) as the within-subjects factor and BDI group (low, high) as the between-subjects factor. Analysis of commission error data confirmed the significant effect of task in this smaller group [*F*_(1, 18)_ = 57.59, *p* < 0.001]. It also revealed a significant effect of group [*F*_(1, 18)_ = 5.39, *p* < 0.05] that was qualified by a significant task by group interaction [*F*_(1, 18)_ = 4.37, *p* = 0.05]. As shown in Figure 1, the significant interaction was due to the high BDI group making significantly more errors of commission than the low BDI group on the SART [*t*_(18)_ = 2.37, *p* < 0.05] whereas the group difference on the high probability task was not significant [*t*_(18)_ = 1.50, *p* = 0.15].

Conducting the continuous analysis for mean RTs demonstrated no significant effect of BDI-II (*F* < 1) but an interaction between task and BDI-II that approached significance [*F*_(1, 41)_ = 3.54, *p* = 0.07], in line with the significant interaction between task and group in the group analysis of RTs, described below. BDI was not found to have a significant influence in the analysis of RTCV [*F*_(1, 41)_ = 2.59, *p* = 0.11] or to interact with task (*F* < 1). Given that omission data were not only unsuitable for parametric analysis but were also unaffected by our task manipulation (see above), we computed the difference in omission errors across the two tasks (difference score = SART omissions minus high probability task omissions); a Spearman correlation indicated the absence of a relationship between this difference score and dysphoric symptoms measured with the BDI-II (*p* = 0.70) and so this was not investigated further.

The group analysis conducted on mean RTs confirmed the significant effect of task in this smaller group [*F*_(1, 18)_ = 33.94, *p* < 0.001] that was qualified by a significant task by group interaction [*F*_(1, 18)_ = 6.20, *p* < 0.05]. The quicker RTs observed on the SART relative to the high probability task were more pronounced in the high BDI [*t*_(9)_ = 9.27, *p* < 0.001] than low BDI [*t*_(9)_ = 1.87, *p* = 0.09] group. The main effect of group was not significant [*F*_(1, 18)_ = 0.31, *p* = 0.58]. Consistent with the RTCV results obtained in the continuous analysis above, there was a significant effect of group [*F*_(1, 18)_ = 5.59, *p* < 0.05], which was due to increased variability in the high BDI group. The effect of task also approached significance [*F*_(1, 18)_ = 1.35, *p* = 0.07], but the interaction between group and task did not [*F*_(1, 18)_ = 1.35, *p* = 0.26]. Thus, as anticipated, RT variability was higher in high BDI participants and there was a trend toward there being greater RT variability in the SART than in the high probability task in this smaller group.

### Influence of dysphoric symptoms on self-report indices of mind-wandering

Whereas the Acting with Awareness scale of the FFMQ provided a measure of the extent to which participants felt they acted with awareness in general, the Thinking Content scale of the DSSQ and positive and negative MW scales provided an index of subjective experience and MW during completion of the laboratory tasks. These measures were conceptualized as trait and state, or dispositional and situational, measures of MW, respectively. The Thinking Content scale provided separate measures of TRI and TUT. Participant data for these measures are provided in Tables 1, 4.

**Table 4:** **Behavioral performance and subjective experience data for participants in the high and low BDI groups**.

	**SART**		**High probability task**	
	**Low BDI (*n* = 10)**	**High BDI (*n* = 10)**	**Low BDI (*n* = 10)**	**High BDI (*n* = 10)**
**BEHAVIORAL PERFORMANCE**				
Mean errors of commission	6.40 (4.32)	11.60 (5.44)	1.4 (1.08)	2.80 (2.74)
Mean errors of omission	0.40 (1.27)	3.90 (7.39)	0.20 (0.63)	2.60 (5.30)
RT	408.25 (96.54)	367.65 (65.36)	440.21 (17.21)	447.29 (57.18)
RTCV	0.21 (0.04)	0.30 (0.10)	0.20 (0.04)	0.26 (0.08)
**SUBJECTIVE EXPERIENCE**				
TRI	1.87 (0.49)	2.74 (0.73)	2.00 (0.58)	2.49 (0.62)
TUT	1.23 (0.40)	1.63 (0.49)	1.29 (0.28)	1.78 (0.80)
Positive MW	1.90 (0.88)	1.70 (0.95)	1.90 (0.99)	1.70 (0.82)
Negative MW	1.70 (0.95)	2.90 (0.57)	1.60 (0.70)	2.50 (0.97)

Data are mean (SD) values.

SART, Sustained Attention to Response Task; BDI, Beck Depression Inventory; RT, Response times; RTCV, RT coefficient of variation; TRI, task-related interference; TUT, task-unrelated thought; MW, mind-wandering.

As noted above, greater severity of dysphoria was associated with lower FFMQ AAS scores, that is, greater self-reported MW in everyday life (*r* = −0.68, *p* < 0.001). Consistent with this pattern, the group analysis demonstrated significantly lower scores on this scale in the high BDI relative to the low BDI group [*t*_(18)_ = −5.35, *p* < 0.001; see Table 1].

In continuous analyses of subjective experience during the SART and high probability tasks, participants with higher BDI scores reported more TUT/TRI overall [*F*_(1, 41)_ = 6.00, *p* < 0.05]. However, BDI did not interact with task (SART vs. high probability task) [*F*_(1, 41)_ = 0.04; *p* = 0.85], thought content (TRI vs. TUT) [*F*_(1, 41)_ = 1.58, *p* = 0.22], or both [*F*_(1, 41)_ = 0.68, *p* = 0.42]. This pattern was replicated in the group analysis where there was a main effect of BDI group [*F*_(1, 18)_ = 9.48, *p* < 0.01], with the dysphoric group reporting more TUT/TRI than the non-dysphoric group. Interactions of BDI group with task [*F*_(1, 18)_ = 1.43, *p* = 0.25], thought content (*F* < 1), or both (*F* < 1) did not approach significance.

With respect to ratings of positive and negative MW, as noted above, participants on average reported greater levels of MW on the SART relative to high probability task, an effect that was due to elevated MW on negative themes. Neither continuous nor group analyses indicated an influence of BDI on MW overall [*F*_(1, 41)_ = 0.79, *p* = 0.38; *F*_(1, 18)_ = 2.06, *p* = 0.17]. However, there was a significant interaction between valence and BDI group [*F*_(1, 18)_ = 13.69, *p* < 0.001]. Whereas levels of negative MW were significantly higher in the dysphoric than in the control participants [*t*_(18)_ = 3.30, *p* < 0.01], levels of positive MW were not found to differ significantly [*t*_(18)_ = 0.54, *p* = 0.60]. The 3-way interaction between task, valence, and participant group, however, was not significant [*F*_(1, 18)_ = 0.24, *p* = 0.62].

### Effects of age on task performance and subjective MW

Although we did not have any a priori hypotheses regarding age, research has sometimes shown that the incidence of depression may be higher in older individuals and furthermore that the incidence of MW may decline in older adults. In the present sample, age did not relate to BDI-II (*r* = 0.17, *p* = 0.27) or to our dispositional measure of MW (*r* = 0.07, *p* = 0.67). However, there was some indication that reports of MW decrease with increasing age following both the SART and the high probability task, respectively, (correlation between age and TUT: *r* = −0.30, *p* = 0.05 and *r* = −0.38, *p* = 0.01, following the SART and high probability tasks, respectively). Given this association, the analyses above were repeated with age entered as a covariate. The pattern of results reported above remained virtually the same in all cases; that is, in no case did a result change from being significant to non-significant or *vice versa*.

### Behavioral and self-report measures of MW and their relation to other participant variables

An average measure of stimulus-independent thought was computed for each participant by taking the average of reports of TRI and TUT. Pearson correlations focused on commission errors for the SART and high probability task as the majority of studies on MW and cognitive failures have emphasized this measure. These are presented in Table 5. Though commission errors on the SART and high probability task were found to correlate highly (*r* = 0.39, *p* = 0.01) and the relationship between subjective MW and commission errors on the SART was significant (*r* = 0.55, *p* < 0.01), the relationship between MW and errors on the high probability task was not (*r* = 0.01, *p* = 0.94). Furthermore, the relationship between errors on the SART and rumination approached significance (*r* = 0.29, *p* = 0.06), whereas this did not achieve significance for the high probability task (*r* = 0.17, *p* = 0.28). No association was found between commission errors and either the AAS “acting with awareness” subscale of the FFMQ or everyday attentional failures measured by the ARCES for either task (*p*s > 0.25). It is worth noting that when these correlations were repeated with only TUT as a measure of MW, the correlations were broadly similar to those reported above for the TUT/TRI composite; however, the relationship between TUT and rumination was not significant (SART: *r* = 0.24, *p* = 0.13; high probability task: *r* = 0.26, *p* = 0.09).

**Table 5:** **Correlations between behavioral performance, self-reported stimulus-independent thought and other participant characteristics (*n* = 43)**.

		**Stimulus-independent thought ^a^**	**AAS**	**ARCES**	**RS**
**SART**					
Commission errors	*r*	0.55	−0.17	−0.03	0.29
	*p*	0.001	0.28	0.84	0.06
Stimulus-independent thought ^a^	*r*		−0.25	0.12	0.35
	*p*		0.10	0.46	0.02
**HIGH PROBABILITY TASK**					
Commission errors	*r*	0.01	−0.04	−0.07	0.17
	*p*	0.94	0.83	0.65	0.28
Stimulus-independent thought	*r*		−0.23	0.12	0.37
	*p*		0.14	0.46	0.02

a Stimulus-independent thought was computed as the average of reported TUT and TRI following completion of the SART or high probability task; AAS, Acting with Awareness Scale; ARCES, Attention-Related Cognitive Errors Scale; RS, Short Response Styles Questionnaire.

## Discussion

This exploratory study investigated whether a task manipulation that has been shown previously to discriminate between individuals with high and low levels of everyday inattentiveness modulates behavioral and self-report indicators of MW, particularly in dysphoric individuals. The two cognitive tasks were go/no-go tasks that require participants to make a response on *go* trials and to withhold a response on critical no-go “target” trials. In the SART, the probability of no-go trials was 11% whereas on a high probability go/no-go task that was matched in every other respect, the probability of no-go targets was 50%. Previous research has shown that a reduced probability of no-go trials increases the tendency to automatic and “mindless” responding and performance has been linked to everyday absentmindedness in the form of cognitive failures in brain-injured populations (Robertson et al., 1997; Manly et al., 1999). Furthermore, performance indices on the SART, typically commission errors and RTCV, have increasingly been conceptualized as behavioral indicators of MW (e.g., Smallwood et al., 2004; McVay and Kane, 2009; Marchetti et al., 2012).

In support of Hypothesis 1 and in line with previous data (Manly et al., 1999), our manipulation of the probability of no-go targets had the intended effect on task performance. Overall, participants made significantly more errors of commission in the SART relative to the high probability task. This replicates previous work showing that it is more difficult to sustain one's attention when there is a low probability of targets (Giambra, 1995; Manly et al., 1999). Also consistent with previous reports, participants had quicker but more variable RTs in the SART relative to the high probability task. It has been suggested that RT variability may reflect subtle differences in RT that are due to lapses of attention (e.g., McVay and Kane, 2009; Seli et al., 2012).

Subjective and retrospective reports of thought content (TUT and TRI) during the SART and the high probability task were indexed using the DSSQ (Matthews et al., 1999, 2002). Additional scales were also administered to index the extent to which participants' minds wandered to positive and negative themes. The data here partially supported Hypothesis 2. Overall, participants reported higher levels of TRI than TUT, an effect that was more evident following the SART than the high probability task. MW was also higher for negative than positive content and higher following the SART than following the high probability task. Rates of positive and (especially) negative MW were further elevated following the SART relative to the high probability task. Thus, behavioral and self-report data are broadly consistent with the idea that the SART can be conceptualized as a laboratory index of mindlessness (e.g., Smallwood et al., 2009). The current study further indicates that this claim can be made with some specificity as behavioral and self-report indicators of MW were elevated in the SART relative to the high probability task. It is worth noting that though a previous investigation examined performance on the SART with a high and low probability of targets (Smallwood et al., 2004) that study additionally manipulated the speed of presentation and did not specifically examine the relationship of their findings to dysphoric mood. Thus, it was not possible to ascertain how target probability interacted with mood status to influence performance and subjective experience.

There was also some support for our third hypothesis that dysphoric individuals would demonstrate difficulties that were either exclusive to or more pronounced on the SART than on the high probability task. Though depression severity was not found to interact significantly with the two tasks in analyses that included BDI as a continuous measure, the analysis of high and low dysphoric individuals confirmed a task by dysphoric group interaction. This interaction was due to dysphoric participants making more errors of commission than the non-dysphoric group on the SART but not the high probability task. Though it has been shown previously that depression is associated with impairments on the SART (Farrin et al., 2003, but see Carriere et al., 2008; Deng et al., 2012), it was not possible to ascertain from those studies whether the observed deficits were due to more general aspects of the task, such as the requirement to withhold a behavioral response, or specifically to the low probability of targets that encouraged a highly repetitive response and tendency to a habitual mode of “mindless” responding (Robertson et al., 1997; Manly et al., 1999). The current findings suggest that these earlier findings (Farrin et al., 2003; Stawarczyk et al., 2012) are unlikely to reflect a general cognitive or behavioral deficit in dysphoric participants and are more likely to relate to difficulties sustaining attention over time that is, maintaining an executive stance over what might otherwise become repetitive and automatic responding. As anticipated, the pattern observed here in dysphoric and non-dysphoric groups resembles that found previously in individuals that differed in everyday self-reported absentmindedness (Manly et al., 1999).

This study furthermore predicted increased subjective reports of MW in dysphoric relative to non-dysphoric participants, particularly in the SART. The first part of this prediction received clear support. Both continuous and group analyses of MW data showed dysphoric, relative to non-dysphoric, participants reporting increased levels of subjective MW (TRI/TUT). However, the effects of BDI were not found to interact with task or type of subjective experience. This indicates that the higher levels of TRI in the SART relative to high probability task were present to an equal extent in high and low BDI individuals. The extent of negative but not positive MW was significantly higher in the dysphoric than non-dysphoric participants but again, the higher levels of negative MW following the SART relative to high probability task did not interact with BDI. These results add to existing findings showing that induced unhappy mood leads to an increase in past-focused MW (Smallwood and O'Connor, 2011). They furthermore suggest that again our modulation had similar effects on MW in the high and low BDI participants. Across the sample, subjective reports of stimulus-independent thought were found to correlate with commission errors on the SART but not on the high probability task. Furthermore, there was a significant correlation between BDI and AAS scores, showing that dysphoric individuals rate themselves as acting with reduced awareness in everyday life, or being less mindful. Given that MW and mindfulness are conceptualized as opposing constructs both here and elsewhere (Mrazek et al., 2012), the correlation between BDI and acting with awareness scores is consistent with dysphoric individuals reporting difficulties with MW in everyday life.

Taken together, these data provide partial support for our hypotheses relating to the subjective experience of MW. Dysphoric participants reported experiencing elevated MW across three different self-report measures—(1) a retrospective measure of TRI/TUT experience during task performance; (2) a retrospective measure of MW on negative but not positive themes; and (3) a trait measure of MW in everyday life. However, though our task manipulation resulted in higher levels of self-reported stimulus-independent thought, and particularly self-reported TRI and negative MW, this was not evident to a greater extent in our dysphoric sample.

Consistent with predictions, the relationship between subjective reports of stimulus-independent thought and commission errors on the SART was significant and there was some indication of a relationship between SART errors and rumination scores. In contrast to expectations, no association was found between SART errors and either the AAS or everyday attentional failures measured by the ARCES. Thus, the associations described in Hypothesis 4 were partially supported. The reason for the failure to find these latter associations is unclear, as previous research conducted in 363 participants demonstrated a reliable association between SART errors and scores on the ARCES and a specifically-derived measure of mindful attention awareness (Smilek et al., 2010), but limited power is likely to have played a role.

Why the effects of our task manipulation on subjective experience were seen primarily for TRI rather than TUT is not apparent, as previous work has shown that it is reports of TUT, in particular that relate to SART performance metrics (e.g., Smallwood et al., 2009; Stawarczyk et al., 2011). TRI incorporates thoughts about the task that are not directed at actual completion and reports are thus considered to be associated with suboptimal performance (Matthews et al., 1999; Stawarczyk et al., 2011). There are further differences between these two types of thought. For example, TUT increases with increasing time on task, whereas TRI does not (Stawarczyk et al., 2011). It has been suggested that TRI may to an extent reflect strategic deployment of attention to the task in response to an attentional lapse (Smallwood et al., 2004). It is unlikely, however, that the present elevated levels of negative MW can be explained solely by higher TRI reports, however, as negative MW, but not specifically TRI (as indicated by the absence of an interaction between BDI and thought content), was elevated in dysphoric relative to non-dysphoric participants.

As this was an exploratory study, it is particularly important to acknowledge its limitations. First, the sample was small and contained a majority of participants with low BDI scores. This meant that applying a median split to the sample would have produced a dysphoric group that included a significant number of participants with BDI-II scores of 8 and above, but 14 is the recommended threshold for “mild depression” on the BDI-II (Beck et al., 1996). Adopting an upper and lower quartile approach instead meant that all participants in the dysphoric group achieved the minimum depression cutoff, but admittedly, the resulting dysphoric and non-dysphoric samples were small. Reliable SART effects have been reported previously in comparably small samples (Manly et al., 1999), but whether this applies to self-report measures of MW is less clear. The current findings would thus benefit from replication in larger samples of clearly defined depressed and non-depressed participants.

Second, assessing MW retrospectively may not be the optimal way to assess the subjective experience of MW during a task. The advantages and disadvantages of these approaches have been discussed elsewhere (e.g., Smallwood and Schooler, 2006). Retrospective reports rely on thought content being available to awareness and memory retrospectively, and this may be particularly problematic where there are difficulties with memory, as can be the case in depression (Johnson and Magaro, 1987; Dalgleish et al., 2007). An alternative is to use “probe” or self-caught methods, which allow the participant to specify the focus of their attention and the presence or absence of MW online. The disadvantage of these approaches, however, is that the prompts can interfere with the very processes that are under investigation and it would have been a challenge to implement these alongside the task manipulation employed here. A third potential limitation is that reports of TUT were low in this study. As it has been suggested that time on task increases MW (Smallwood et al., 2003), lengthening the duration over which participants are required to sustain their attention could be beneficially applied here.

A final issue is the extent to which behavioral performance on the SART is a good behavioral, objective, index of MW. Whereas some investigators have argued that the SART requires sustained attention to response rather than a response inhibition capacity (e.g., Manly et al., 1999), others have argued that the SART places high response inhibition, and not necessarily sustained attention, demands on participants (e.g., Carter et al., 2013). It is thus possible that any requirement for response inhibition could be higher in the SART than the high probability task, due to the lower frequency of no-go targets. Consequently, the poorer relative performance on the SART observed in our dysphoric participants could be due, at least in part, to difficulties with response inhibition—an interpretation that could explain why dysphoria was found to predict differences in performance, but not self-report MW, across the two tasks. In this respect, it is worth noting, first of all that depression is not routinely associated with deficits in inhibitory control over behavioral responding (e.g., Murphy et al., 1999), and second of all that there are possible alternate explanations for this difference across measures. For instance, the reports of depressed individuals may not accurately reflect MW episodes due to reduced meta-awareness or alternatively, difficulties with memory that are frequently associated with depression (Ramponi et al., 2004), as noted above. Future empirical work is required to ascertain the relative contributions of these different processes and their relationship to dysphoric mood.

Despite these limitations, our findings provide encouraging preliminary support for the use of this task manipulation (low vs. high probability of no-go targets) as one that modulates behavioral and self-report indices of MW in dysphoric individuals. Across participants, errors of commission were elevated in the SART relative to the high probability task, and this effect was particularly evident in our dysphoric participants. With respect to subjective indicators, reports of stimulus-independent thought in the form of TRI and negative MW were elevated in the SART relative to the high probability task, though this modulation was not present to a greater extent in our dysphoric sample. Dysphoric participants did, however, report elevated self-reported MW across retrospective self-report and everyday dispositional measures of MW. The evidence base is beginning to converge on the idea that mood and MW can have a reciprocal influence on one another, and that interventions like MBCT may work, at least in part, by training individuals to be present in the moment. Given this, the application of a method that can modulate both behavioral and self-report levels of stimulus-independent thought in the laboratory, not only in healthy participants, but also in those experiencing significant depression, could be useful both theoretically and clinically. At the same time, it is important to bear in mind that MW can be both constructive and unconstructive, depending upon the precise circumstances. Smallwood and Andrews-Hanna (2013) have recently proposed a framework in which they highlight both task context and thought content as critical factors that determine the costs and benefits of stimulus-independent thought. An approach along the lines of that used here might be useful in allowing future investigators to examine the contributions of context and content simultaneously.

It is our view that developing a clearer understanding of the relationship between MW and low mood will ultimately suggest ways in which clinical interventions can best target MW to help treat depression and related conditions. Specification of the precise nature of this relationship could have important implications for our understanding and delivery of existing clinically-derived interventions for depression, such as MBCT, as described above, or Behavioral Activation (BA) (Martell et al., 2013). BA is a cognitive-behavioral intervention in which individuals must keep meaningful plans, goals, and activities in mind such that they can guide and direct subsequent behavior. It is relevant here that MW has also been shown to be linked to goal maintenance that is, the ability to maintain behavior in a goal-directed manner (McVay and Kane, 2009), and that this ability may be compromised in depression (Dalgleish et al., 2007).

Future studies that address these limitations in a larger and more clearly specificed sample of depressed and non-depressed individuals will allow fine-tuning of the present methodology. The obvious next step would be to investigate how a more reliable and effective modulation of MW impacts upon mood state. This type of approach has been applied previously, by manipulating the rate of stimulus presentation. Teasdale and Rezin (1978) demonstrated that a slow, relative to fast, rate of presentation of information led to an increase in the number of negative thoughts and lower mood in clinically depressed individuals. Another avenue for future investigation, on the basis of the present findings, is to explore whether cognitive training that focuses on increasing the ability to sustain attention could increase control over everyday MW and benefit mood. An approached based in the techniques used here has already been translated into clinical practice in brain-injured populations, where the relationship between performance on the SART and everyday absentmindedness has been specified alongside factors that modulate these (Robertson et al., 1997; Manly et al., 1999). In particular, this research has provided the basis for an intervention that delivers periodic “content-free” auditory cues that are intended to remind patients to refocus on the current task in both laboratory and real world settings (Manly et al., 2002, 2004; Levine et al., 2011). It is possible that refinement of the current methodology could lead to the development of a similar type of intervention in depressed individuals that could be beneficial in terms of improving low mood.

### Conflict of interest statement

The authors declare that the research was conducted in the absence of any commercial or financial relationships that could be construed as a potential conflict of interest.
